# Integrative analysis of shared genetic pathogenesis by autism spectrum disorder and obsessive-compulsive disorder

**DOI:** 10.1042/BSR20191942

**Published:** 2019-12-23

**Authors:** Dongbai Liu, Hongbao Cao, Kamil Can Kural, Qi Fang, Fuquan Zhang

**Affiliations:** 1 Department of Neurology, The First People’s Hospital Affiliated to Soochow University, Suzhou, Jiangsu Province, 215006, China; 2 Department of Neurology, Jiangyin People’s Hospital, Jiangyin, Jiangsu Province, 214400, China; 3 Department of Psychiatry, First Hospital/First Clinical Medical College of Shanxi Medical University, Taiyuan, Shanxi Province, 030001, China; 4 Department of Genomics Research, R&D Solutions, Elsevier Inc., Rockville, MD 20852, U.S.A.; 5 School of Systems Biology, George Mason University, Fairfax, VA 22030, U.S.A.; 6 Department of Psychiatry, The Affiliated Brain Hospital of Nanjing Medical University, Nanjing, Jiansu Province, 210029, China

**Keywords:** autism spectrum disorder, gene expression analysis, Network connectivity analysis, Obsessive-compulsive disorder

## Abstract

Many common pathological features have been observed for both autism spectrum disorders (ASDs) and obsessive-compulsive disorder (OCD). However, no systematic analysis of the common gene markers associated with both ASD and OCD has been conducted so far. Here, two batches of large-scale literature-based disease–gene relation data (updated in 2017 and 2019, respectively) and gene expression data were integrated to study the possible association between OCD and ASD at the genetic level. Genes linked to OCD and ASD present significant overlap (*P*-value <2.64e-39). A genetic network of over 20 genes was constructed, through which OCD and ASD may exert influence on each other. The 2017-based analysis suggested six potential common risk genes for OCD and ASD (*CDH2, ADCY8, APOE, TSPO, TOR1A*, and *OLIG2*), and the 2019-based study identified two more genes (*DISP1* and *SETD1A*). Notably, the gene *APOE* identified by the 2017-based analysis has been implicated to have an association with ASD in a recent study (2018) with DNA methylation analysis. Our results support the possible complex genetic associations between OCD and ASD. Genes linked to one disease are worth further investigation as potential risk factors for the other.

## Introduction

Autism spectrum disorders (ASDs) are common, highly heritable neuro-developmental conditions characterized by language impairments, social deficits, and repetitive behaviors. So far, many articles have reported that a number of core pathological features of ASD are also commonly observed in obsessive-compulsive disorder (OCD) [1,2], and similar brain abnormalities have also been suggested between ASD and OCD patients [3]. Additionally, considerable amounts of evidence demonstrate that patients with ASD are at an increased risk of comorbid anxiety disorders [4–7]. For instance, van Steensel et al. [8] reported that approximately 40% of patients with ASD are assigned at least one comorbid diagnosis of anxiety and approximately 17% of children with ASD meet criteria for OCD. Diagnostic characteristics of these patients include persistent and distressing thoughts and behaviors used to ‘cope with’ those thoughts.

In recent years, genetic studies using both genome-wide association study (GWAS) and gene expression data have revealed hundreds of genes associated with both ASD and OCD [9–12]. However, as far as we know, there has been no systematic study performed to investigate the common genes between both diseases.

In the present study, we integrated gene expression data and large-scale literature knowledge database to study the association between OCD and ASD at the genetic level, with the purpose to gain a better understanding of the possible common genetic basis and to identify novel common genes associated with both diseases. The disease-related genes were identified using Pathway Studio (http://www.pathwaystudio.com/), which has been widely used to study modeled relationships between proteins, genes, complexes, cells, tissues, and diseases [13] (http://pathwaystudio.gousinfo.com/Mendeley.html). Updated weekly, the Pathway Studio possesses the largest database among known competitors in the field [14].

## Methods

The large-scale literature data based ASD-gene and OCD-gene relations were studied targeting the identification of common genes associated with both diseases. These disease-implicated genes were then tested using two ASD expression datasets to discover possible novel common genes. After that, functional network analysis was conducted to study the pathogenic significance of the identified genes in ASD.

To validate the stability of the proposed workflow, we analyzed two batches of literature data (updated in August 2017 and March 2019, respectively) compared their results. For the 2017-batch-based analysis, all results were organized in a databased **ASD_OCD**. For the 2019-batch-based analysis, it is in **ASD_OCD_2019.** The downloadable format of these two databases is available at gousinfo.com/database/Data_Genetic/ASD_OCD.xlsx and http://gousinfo.com/database/Data_Genetic/ASD_OCD_2019.xlsx, respectively. The two files are also available as Supplementary files: ASD_OCD.xlsx and ASD_OCD_2019.xlsx.

### Disease–gene relation data

Disease–gene relation data for both ASD and OCD were acquired through large-scale literature data analysis assisted by Pathway Studio (www.pathwaystudio.com) and presented in **ASD_OCD and ASD_OCD_2019.** Besides the full lists of genes linked to both diseases, we also presented the information of supporting references for each disease–gene relation (**ASD_OCD and ASD_OCD_2019: Ref for OCD/ASD Related Genes**), including titles of the references and the related sentences where the disease–gene relationship was identified. The information could be used to locate a detailed description of how a candidate gene is associated with OCD and/or ASD.

### ASD expression data

Two ASD expression datasets were acquired from Illumine BaseSpace Correlation Engine (http://www.illumina.com). After the initial search with target set as ‘Autism Spectrum Disorders’, the expression datasets were screened by the following criteria, including: (1) the data organism is *Homo sapiens*; (2) the data type is RNA expression; (3) the samples of studies come from brain tissues; and (4) the studies are limited to ASD case vs. healthy control study (almost equal number of cases and controls). The top two gene expression datasets (GSE28521: 39 ASD *vs.* 40 healthy controls; GSE38322: 18 ASD *vs.* 18 healthy controls) were selected to test the genes linked to OCD but not with ASD. For a gene to be tested, one-way ANOVA was performed to compare the expression of this gene between ASD controls and cases. The genes that passed an FDR corrected (q = 0.05) were identified as significant potential ASD target genes for further analysis.

### Shorted-path analysis of the target risk genes

For the significant genes identified through expression analysis described above, shorted-path-based network analysis was conducted between the target genes and the disease (ASD/OCD) to identify potential biological connections. The analysis was performed using the ‘Shortest Path’ module of Pathway Studio (www.pathwaystudio.com).

### Protein–protein interaction analysis

To explore the relationships between any identified potential common genes for ASD and OCD, we conducted a literature-based protein–protein interaction analysis (PPI). Two genes were identified to have an association if they have been reported as such in one or more scientific reports. The underlying hypothesis is that, if these genes were associated with both diseases, they might present functional associations between each other.

## Results

### Common genes for OCD and ASD

**Figure 1:**
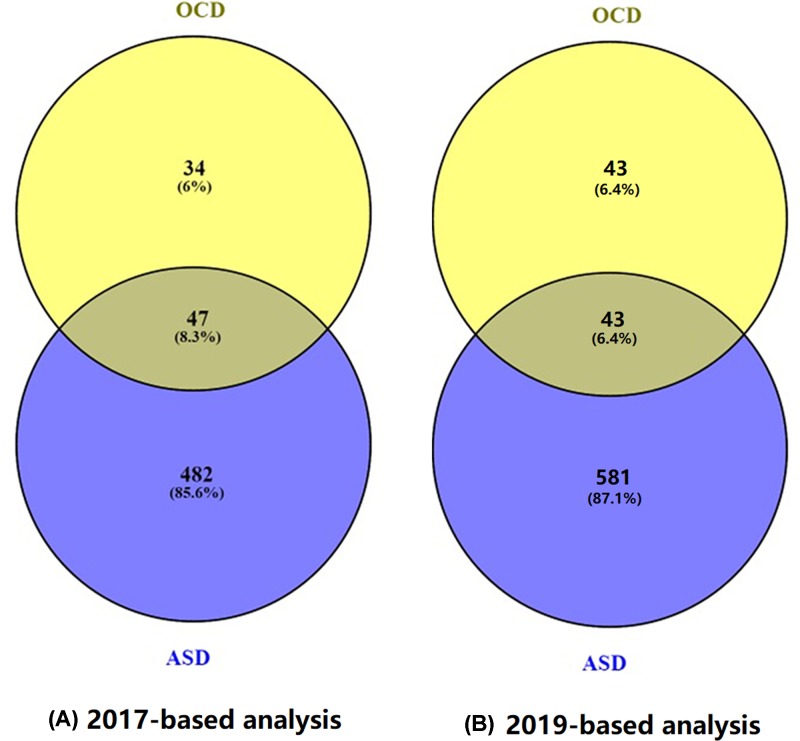
Venn diagram between ASD-genes and OCD-genes (**A**) Results based on the 2017-updated database. (**B**) Results based on the 2019-updated database.

Within the curated **ASD_OCD** database (2017-based-analysis), there were 81 genes associated with OCD, supported by 450 scientific references from 1992 to 2016 (see ASD_OCD**: OCD Related Genes** and **Ref for OCD Related Genes**). For ASD, there were 529 related genes supported by 2098 references from 2000 to 2017 (**ASD_OCD: ASD Related Genes** and **Ref for ASD Related Genes**). A significant overlap of 47 genes was identified for both diseases (Right tail Fisher’s Exact test, *P*-value = 1.66e-48), as shown in Figure 1. The *P*-value here means that, for two random gene sets with the size of 81 and 529, respectively, the probability that they present an overlap of 47 or more is less than 1.66e-48. More information on these 47 genes is presented in **ASD_OCD→47 common genes.**

Within the **ASD_OCD_2019** database (2019-based-analysis), there were 86 OCD genes and 624 ASD genes, with an overlap of 43 genes. The decrease in the common genes was due to the removal of the references of low confidence levels in the new analysis. In this case, the overlap presents a significance *P*-value = 2.64e-39. Please refer to ASD_OCD_2019 for the relevant information.

To test the functional profile of the 47 common genes associated with both ASD and OCD, we conducted a Gene Set Enrichment Analysis (GSEA) using Pathway Studio, with the 47 genes as input for the GSEA. The ten most significantly enriched pathways (*P*-value <8.37E-22, q = 0.05 for FDR) are presented in Table 1. The full 115 pathways/gene sets enriched with *P*-value <3.26e-10, including 46 out of 47 genes, were presented in **ASD_OCD→Common Pathways**. GSEA approach revealed 19 pathways/gene sets (38 unique genes) related to neurosystem, 5 (22 unique genes) to brain function development, 9 (23 unique genes) to behavior and 13 (14 unique genes) to the neurotransmitter. For detailed information regarding these significantly enriched pathways, please refer to **ASD_OCD→Common Pathways**. Our results suggest that OCD and ASD share multiple genetic pathways, through which these 47 genes play roles affecting the pathogenic development of both diseases. The relative newly updated information can be seen in ASD_OCD_2019→Common Pathways, which only presents a minor change in terms of enriched pathways. As shown in Table 1, the top ten pathways by the 43 common genes from the 2019 update presented an overlap of nine pathways with that of the 2017 update. The only different one was the ‘memory’ pathway, which replaced the ‘trans-synaptic signaling’ pathway from the 2017 update.

**Table 1:** Genetic pathways enriched with 47 genes linked to both OCD and ASD

	Name	# of entities	Overlap	Percent overlap	*P*-value	Jaccard similarity
2017 updated database	GO: behavior	759	32	4	1.35E-32	0.041344
	GO: regulation of neurotransmitter levels	339	23	6	3.89E-26	0.063361
	GO: synaptic signaling	387	23	5	4.29E-25	0.055961
	*GO: trans-synaptic signaling*	*387*	*23*	*5*	*4.29E-25*	*0.055961*
	GO: cell–cell signaling	741	27	3	4.82E-25	0.03548
	GO: learning or memory	339	22	6	7.56E-25	0.06044
	GO: signaling	815	27	3	4.64E-24	0.032335
	GO: cognition	379	22	5	6.37E-24	0.054455
	GO: regulation of secretion	985	27	2	4.45E-22	0.026866
	GO: regulation of secretion by cell	888	26	2	8.37E-22	0.028603
2019 updated database	GO: behavior	759	26	3	8.44E-24	0.033505
	GO: regulation of neurotransmitter levels	339	21	6	9.71E-24	0.058172
	GO: learning or memory	339	21	6	9.71E-24	0.058172
	GO: regulation of secretion	985	27	2	4.6E-23	0.026973
	GO: cognition	379	21	5	6.26E-23	0.052369
	GO: regulation of secretion by cell	888	26	2	8.1E-23	0.028729
	GO: cell–cell signaling	741	24	3	1.12E-21	0.031579
	GO: signaling	815	24	2	9.14E-21	0.028777
	*GO: memory*	*162*	*16*	*9*	*9.14E-21*	*0.084656*
	GO: synaptic signaling	387	19	4	9.42E-20	0.046229

For each pathway/Go term, the *P*-value was calculated using Fisher-exact test against the hypothesis that a randomly selected gene group of the same size (47) can generate a same or higher overlap with the corresponding pathway/Go term. All these pathways/Go terms passed the FDR correction (q = 0.05).

### Possible co-regulation between OCD and ASD

**Figure 2:**
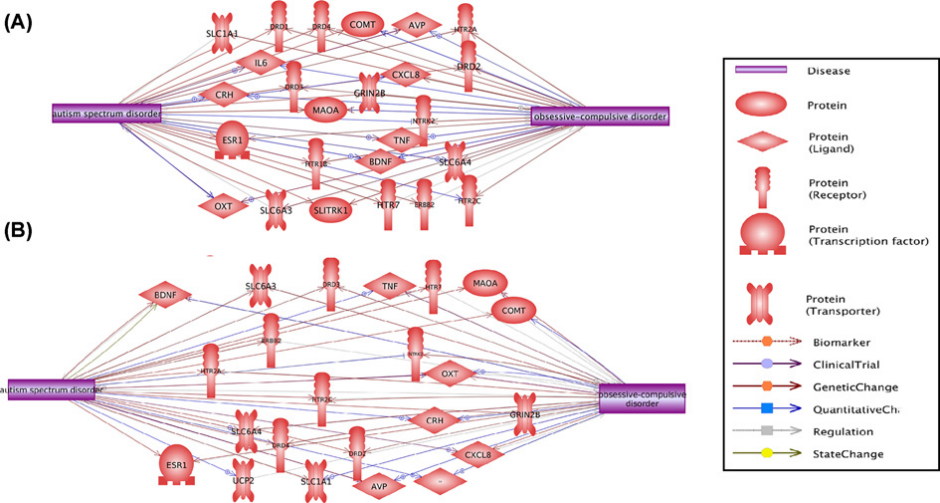
Co-regulation Network between ASD and OCD (**A**) Results based on the 2017-updated database. (**B**) Results based on the 2019-updated database.

2017-based analysis using PS showed that 25 out of the 47 common genes present both downstream and upstream regulation relationships with both ASD and OCD (influenced by and influencing both OCD and ASD), as shown in Figure 2A. The detailed information of the network presented in Figure 2A can be found in **ASD_OCD→** Co-Regulation Network, including the type of the relationship, supporting references, and related sentences from the references where the relationship has been identified.

For 2019-batch related analysis, 23 out of 43 common genes present both downstream and upstream regulation relationships with both ASD and OCD, as shown in Figure 2B. More information is presented in **ASD_OCD_2019→** Co-Regulation Network. Figure 2 showed that OCD and ASD might influence the pathogenic development of each other through these genetic networks.

### Gene expression analysis

**Figure 3:**
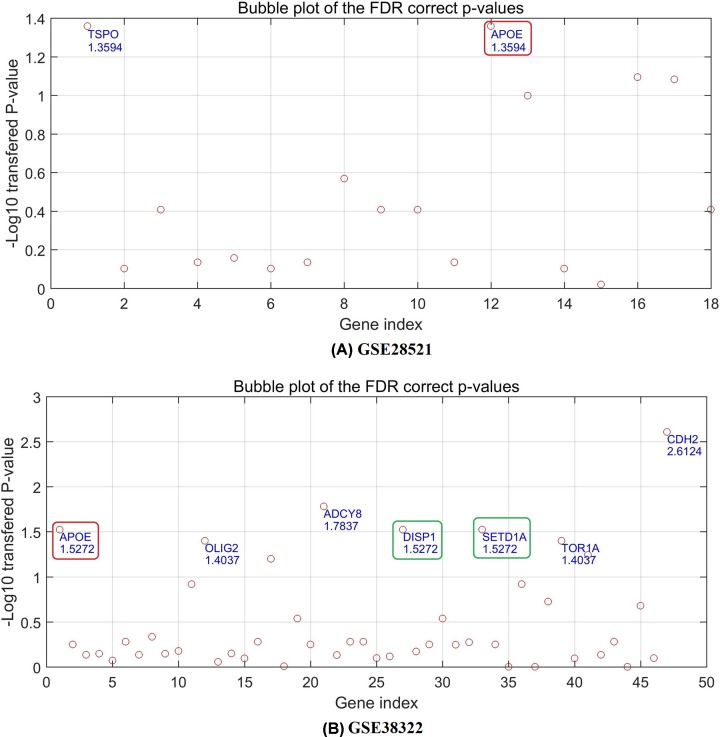
The *P*-values of the OCD individual genes for ASD case/control expression comparison in dataset GSE28521 and GSE38322 (**A**) The *P*-values of the OCD-specific genes in dataset GSE38322. (**B**) The *P*-values of the OCD-specific genes in dataset GSE38322. The *P*-values have been through FDR correction with q = 0.05 and logic transformation using ‘−log10’. Names and corresponding transferred *P*-values of selected genes passed the FDR correction (q = 0.05) were marked at corresponding positions. The two genes (*DISP1* and *SETD1A*) highlighted by green circle were newly identified by the 2019-based analysis, and the red-circle highlighted gene (*APOE*) was replicated in a 2018-published article.

Although there was a significant overlap between ASD-genes and OCD-genes, some genes were linked to one disease only. Specifically, from ASD_OCD, there are 34 genes linked to OCD but not to ASD, this number changed to 43 for ASD_OCD_2019. Here we tested the correlation between these OCD-specific genes and ASD, using expression data (GSE28521 and GSE38322). Figure 3 elucidates the ‘−log10’ transferred *P*-values (q = 0.05 for FDR) of each gene tested. The detailed results are presented in ASD_OCD and ASD_OCD_2019 (GSE28521 and GSE38322, respectively), including the *P*-values and FDR correction status.

Figure 3 showed that two genes (*TSPO* and *APOE*) from GSE28521 passed the *P*=0.05 FDR (Figure 3A), and seven genes (*CDH2, ADCY8, APOE, TOR1A, OLIG2, DISP1*, and *SETD1A*) from GSE38322 passed the FDR. To note, two genes (*DISP1* and *SETD1A*) were newly identified in the 2019-based analysis, and the gene *APOE* was replicated by a recently published study [15], which showed that APOE presented a significant association with ASD in DNA methylation analysis. The replication demonstrated the effectiveness of the workflow proposed in the present study.

### PPI and shorted-path analysis

**Figure 4:**
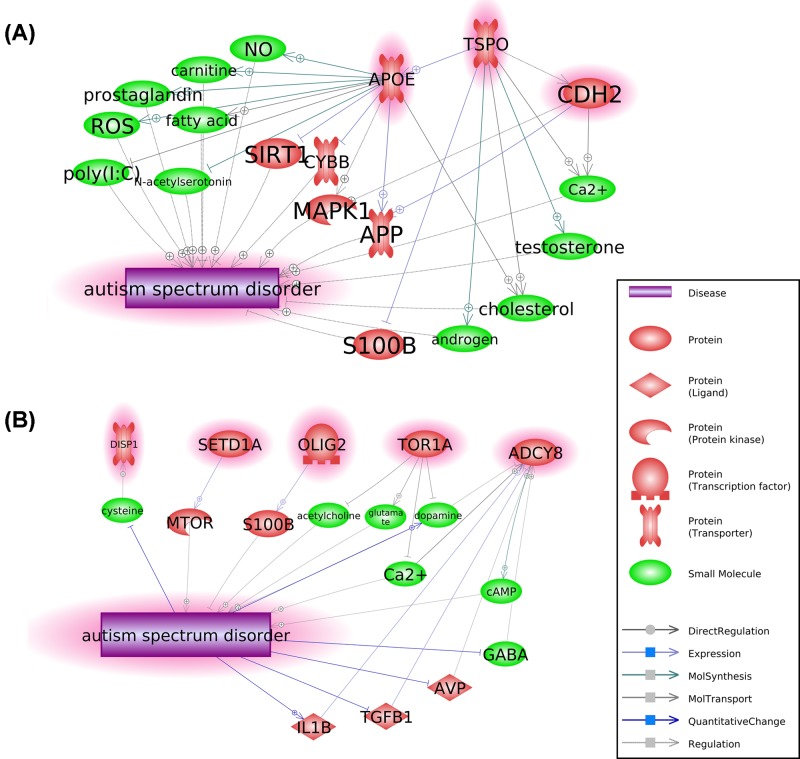
PPI and shortest path analysis results (**A**) Three connected genes (*APOE, TSOP*, and *CDH2*) present association with ASD. (**B**) Five disconnected genes (*DISP1, SETD1A, OLIG2, TOR1A*, and *ADCY8*) present association with ASD.

By using a shorted-path approach (conducted by using Pathway Studio), we explored possible pathways among the eight identified genes and ASD, as shown in Figure 4. The shorted path analysis was conducted to identify entities (e.g., drugs and proteins) that were linked to both gene and ASD in a directed path (e.g., ADCY8→BDNF→ASD). The detailed information of the relationships in Figure 4 is presented in **ASD_OCD_2019→**Shortest_Path.

## Discussion

Previous studies showed that OCD is closely related to ASD [1–3]. In the present study, we integrated large-scale literature-based relation data and gene expression data to test the hypothesis that ASD and OCD display a significant shared genetic basis in terms of common related genes. Gene expression data analysis suggested novel potential commonly genes for both diseases, supported by the functional network analysis. To note, we used literature data from both 2017 and 2019 to generate solid results using the proposed workflow.

Genes linked to OCD and ASD reveals significant overlaps (see Figure 1, *P*-value = 6.76e-34 and *P*-value = 2.64e-39 for the 2017- and 2019-based analyses, respectively). Pathway analysis showed that the common genes were significantly enriched in the pathways that were implicated with both ASD and OCD, such as the synaptic transmission, dopaminergic pathway (GO: 0001963) [16,17], the dopamine neurotransmitter receptor activity pathway (GO: 0004952) [18,19], memory (GO ID: 0007613) [20,21] and behavioral fear response (GO: 0001662) pathways [22,23]. These results suggest that OCD and ASD share multiple genetic pathways. Through these pathways, a large group of genes influences the pathogenic development of both diseases. The shared genetic commonality partially explains the observation that a number of core pathological features of ASD are commonly observed in OCD [1,2], and similar brain abnormalities have also been suggested between both ASD and OCD patients [3].

Moreover, we observed a co-regulation network between OCD and ASD, composed of more than 20 genes (Figure 2). The genes within the network are downstream targets of ASD/OCD, while they are also the upstream regulators of OCD/ASD. Our findings support the genetic association between OCD and ASD.

To explore the possible linkage between the genes that have only been implicated with OCD but not ASD, we used two ASD gene expression datasets (GSE28521 and GSE38322) to explore the OCD-specific genes in case of ASD. For the 2017-based analysis, results revealed six OCD genes also present significant differences (FDR corrected *P*-value<0.05) between ASD cases and healthy controls, including two genes (*TSPO* and *APOE*) from GSE28521 and five genes (*CDH2, ADCY8, APOE, TOR1A*, and *OLIG2*) from GSE38322. Notably, the gene *APOE*, that has been identified in both datasets, in the 2017-based analysis was reported to have a potential association with ASD in a recent study [15], with supported the effectiveness of the proposed workflow. It has been shown that APOE methylation in pediatric patients with ASD was significantly higher than that in the healthy controls (median PMR, 33 vs. 11%; *P*=2.36 × 10^−10^). Thus, APOE hypermethylation in peripheral blood DNA may be used as a diagnostic biomarker for ASD. In addition, the 2019-based analysis suggested two more common genes for both OCD and ASD (*DISP1* and *SETD1A*), as highlighted by the green circle in Figure 3B.

Functional network analysis showed that these eight OCD genes also presented functional correlation with ASD, forming a genetic network supported by over 1600 scientific reports (Figure 4; see **ASD_OCD_2019:** Shortest_Path). These results suggested multiple genetic paths through which these genes play roles for the pathological development of ASD.

PPI showed that TSPO regulates both APOE and CDH2, and these three genes presented multiple common pathways regulating ASD (Figure 4A). On the other hand, we see no connection between the rest five genes (Figure 4B). Based on the hypothesis that, if two genes play roles within OCD and ASD, they were more likely functionally linked to each other than not. Thus, our results suggested that more attention should be paid to the three genes (*TSPO, APOE*, and *CDH2*).

Specifically, the suggested potential APOE–ASD association has also been proposed by a recent study [15]. APOE is a widely studied and well-known gene, primarily produced by the liver and macrophages, and mediates cholesterol metabolism in an isoform-dependent manner. APOE is the principal cholesterol carrier in the brain [24], with three major alleles: APOE2 (Cys^112^, Cys^158^), APOE3 (Cys^112^, Arg^158^), and APOE4 (Arg^112^, Arg^158^) [25]. The APOE4 variant was frequently reported to be the largest known genetic risk factor for late-onset sporadic Alzheimer’s disease [26]. The genetic paths found in the functional network connecting APOE with ASD (Figure 4A) may provide new insights for a possible linkage between APOE and ASD. For instance, APOE is associated with the production of NO in macrophages [27], while increased NO synthase plays a role in the pathologic development of ASD [28]. APOE can also significantly inhibit RELN binding [29], while RELN has been reported to play an important pathophysiological mechanism in ASD [30]. These findings suggested a clue for the possible association between APOE and ASD. More of these genetic paths can be identified from **ASD_OCD_2019_Shortest_Path**.

To note, the ASD and OCD related genes employed in the present study were identified from a literature review. However, a reported relationship in the publication does not necessarily guarantee a true biological gene–disease linkage. Therefore, findings from the present study are more suggestive than confirmative. Further study, including biological experiments, is preferred to confirm the results identified in the present study. In addition, the results of our study guaranteed further study to test the ASD specific genes in the case of OCD. Further study using more datasets and direct results from biological experiments is preferred to confirm the results identified in the present study.

## Conclusion

The results from the present study support the hypothesis that OCD and ASD present significant association at the genetic level, which may explain their common pathological features in the clinic. Additionally, eight genes were suggested as common genes for both OCD and ASD, and one of them has been recently confirmed by another study. To our knowledge, this is the first study to integrate large-scale literature relation data and gene expression data for a systematical evaluation of the associations between OCD and ASD at the genetic level. Findings here may add new insights into the current field of OCD–ASD correlation study, and guarantee further studies using more datasets to test novel potential risk genes for both ASD and OCD.

## Availability of Data and Materials

All the data used in the present study are provided in two online cross-disease genetic databases: ASD_OCD_2019 and ASD_OCD.

## Abbreviations

ASD: autism spectrum disorder
GSEA: gene set enrichment analysis
OCD: obsessive-compulsive disorder
PPI: protein–protein interaction analysis
